# Biogenic Selenium Nanoparticles: Anticancer, Antimicrobial, Insecticidal Properties and Their Impact on Soybean (*Glycine max* L.) Seed Germination and Seedling Growth

**DOI:** 10.3390/biology12111361

**Published:** 2023-10-24

**Authors:** Asmaa Abdelsalam, Heba El-Sayed, Heba M. Hamama, Mostafa Y. Morad, Abeer S. Aloufi, Rehab M. Abd El-Hameed

**Affiliations:** 1 Botany and Microbiology Department, Faculty of Science, Helwan University, Helwan 11795, Egypt; asmaa_abdulsalam@science.helwan.edu.eg (A.A.); drhebaelsayed39@gmail.com (H.E.-S.); roby1432000@gmail.com (R.M.A.E.-H.); 2 Entomology Department, Faculty of Science, Cairo University, Giza 12613, Egypt; hahmed@sci.cu.edu.eg; 3 Zoology and Entomology Department, Faculty of Science, Helwan University, Helwan 11795, Egypt; mustafa_ali@science.helwan.edu.eg; 4 Department of Biology, College of Science, Princess Nourah bint Abdulrahman University, P.O. Box 84428, Riyadh 11671, Saudi Arabia

**Keywords:** antifungal, antibacterial, MCF-7 breast cancer, cytotoxicity, soybean, *Spodoptera littoralis*

## Abstract

**Simple Summary:**

The standard approaches used in the fields of cancer therapies, microbial infection mitigation, and agricultural improvement exhibit intrinsic limitations, especially in terms of their efficacy, precision, expenses, and environmental impacts. Biogenic nanoparticles, on the other hand, offer eco-friendly, multifunctional solutions in numerous disciplines. The present work highlights the crucial importance of biogenic SeNPs across various fields, emphasising their potential in the realms of agriculture, cancer treatment, and infection control. The inhibitory effect of SeNPs on MCF-7 breast adenocarcinoma cells, coupled with antibacterial activity against *Proteus mirabilis* and *Klebsiella pneumoniae*, underscores SeNPs’ potential for cancer therapy and microbial control. In agriculture, the positive effect of SeNPs on the germination dynamics and seedling development of soybean, a crucial economic plant, reveals their potential for enhancing agricultural yield. SeNPs effectively controlling the cotton leafworm and inhibiting plant pathogenic fungi are underscored in sustainable agriculture, as they provide a bio-based strategy for managing pests and preventing plant diseases. The present study provides opportunities for further investigation, promoting the examination of strategies to enhance the utilisation of SeNPs in the fields of cancer treatment, agriculture, and infection management. Valuable directions encompass the refinement of the synthesis process to optimise characteristics, the investigation of supplementary biological effects, and the evaluation of long-term environmental implications.

**Abstract:**

Selenium nanoparticles (SeNPs) have demonstrated significant potential in a variety of disciplines, making them an extremely desirable subject of research. This study investigated the anticancer and antibacterial properties of my-co-fabricated selenium SeNPs, as well as their effects on soybean (*Glycine max* L.) seeds, seedling growth, cotton leafworm (*Spodoptera littoralis*) combat, and plant pathogenic fungi inhibition. SeNPs showed anticancer activity with an IC_50_ value of 1.95 µg/mL against MCF-7 breast adenocarcinoma cells. The myco-synthesized SeNPs exhibited an antibacterial effect against *Proteus mirabilis* and *Klebsiella pneumoniae* at 20 mg/mL. The use of 1 µM SeNPs improved soybean seed germination (93%), germination energy (76.5%), germination rate (19.0), and mean germination time (4.3 days). At 0.5 and 1.0 µM SeNPs, the growth parameters of seedlings improved. SeNPs increased the 4th instar larval mortality of cotton leafworm compared to control, with a median lethal concentration of 23.08 mg/mL. They inhibited the growth of *Fusarium oxysporum*, *Rhizoctonia solani*, and *Fusarium solani*. These findings demonstrate that biogenic SeNPs represent a promising approach to achieving sustainable progress in the fields of agriculture, cancer therapy, and infection control.

## 1. Introduction

The overuse of synthetic chemicals such as antimicrobials, insecticides, and plant fertilizers is linked to negative effects on human health, pollutes the environment, disrupts natural ecosystems, and ultimately can lead to the evolution of pests and pathogens that are resistant to these chemicals [1,2]. Consequently, there is a pressing need for the development of safer and less harmful strategies for pest and microbial disease control and plant fertilizer, as well as for the sustainable use of existing methods [3,4,5].

Nanotechnology is one of the more recent inter-disciplinary fields that has made significant contributions to a variety of applications [6]. Nanobiotechnology is a subfield of nanotechnology concerned with the production and manipulation of nanoparticles using living organisms [7]. Microbial synthesis of nanoparticles is regarded as an eco-friendly method that can aid in sustainable synthesis and is economically viable, which supports the increasing use of nanoparticles in a variety of applications [4]. Fungi are among the species that contribute to the green fabrication of nanoparticles and have many advantages over other organisms [8]. Their nanoparticle production is eco-friendly and scalable due to their adaptability, tolerance of high metal concentrations, and abundance of molecules capable of converting metal ions into nanoparticles [9]. One of the most prevalent and ubiquitous genera in the fungi kingdom is *Penicillium*. Species in the genus *Penicillium* are responsible for a rich diversity of essential secondary metabolites with major medical and commercial applications [10]. Numerous species in the genus *Pencillium* are utilized in the production of nanoparticles, like *Penicillium expansum*, *Penicillium crustosum*, and *Penicillium verrucosum* [11,12,13]. Selenium nanoparticles (SeNPs) have been documented to possess significant antibacterial activity, rendering them a prospective candidate for inhibiting the growth of antimicrobial drug-resistant microbes [14].

Although selenium is necessary in the diet as a trace element for proper health and development, high dietary intakes of this element can be toxic. Alternatively, selenium nanoparticles demonstrated several features, such as high stability, bioactivity, and low toxicity, and had superior absorption in long-term supplementation compared to selenium [15]. The vast bulk of selenium nanoparticles are synthesized using microbes such as bacteria and fungi [16,17]. Selenium nanoparticles have been reported as promising substances with various therapeutic applications. The antioxidant activity of selenium nanoparticles has been reported [18]. Selenium nanoparticles have been shown to have anticancer properties against human melanoma and glioblastoma cells [19], as well as the ability to inhibit colon cancer cells [16].

Selenium nanoparticles have recently been developed for use in the agricultural field, specifically as plant fertilizer to boost germination rates, increase crop yields and productivity, and help plants better withstand and adapt to adverse conditions; they also have fungicidal and insecticidal properties [20]. *Glycine max* (soybean) is an annual legume belonging to the Fabaceae family. For thousands of years, it has been recognized as one of the most crucial crops worldwide [21]. It contributes to soil fertility and is known to contain a significant amount of proteins, oils, and polysaccharides of high quality [22]. Numerous industrial food products like soymilk, soy paste, and soy sauce are derived from soybeans [23]. Two of the most essential early phases in the life and development of most crops are the germination of seeds and the subsequent growth of seedlings [24,25]. Soybean germination and yield can be influenced by various factors, including temperature [26], and the presence of *Fusarium* spp. infection, which can result in the suboptimal growth and decay of soybean seedlings [27]. Soybean plants are also attacked by *Spodoptera littoralis* [28], which causes plant defoliation and reduces plant yield.

*Spodoptera littoralis* (Boisd.) (Lepidoptera: Noctuidae), also known as the cotton leafworm, is one of the most economically damaging insects in Egypt’s agricultural areas. It is classified as a severe insect because it causes significant economic damage to cotton yield, among other crops and vegetables [29]. The main problems in controlling this pest relate to increasing pest resistance to chemical insecticides and environmental contamination by multiple applications of insecticides to attain a satisfactory control level [30].

The present study aims to explore the diverse impact of the myco-fabricated SeNPs and elucidate their potential comprehensive utility as anticancer and antibacterial agents in addition to their impact on the germination and growth of soybean seeds and their impact on cotton leafworm and plant pathogenic fungi control.

## 2. Materials and Methods

### 2.1. The Fungal Strain

The synthesis of SeNPs was performed by *Penicillium chrysogenum*. This organism was collected and identified, and its genome was deposited in the GenBank under the accession number MZ945518. *Penicillium chrysogenum* was grown on a solidified agar medium containing saja peptone, yeast extract, malt extract, glucose, and NaCl [31].

### 2.2. Synthesis of SeNPs

*P. chrysogenum* was subjected to frequent sub-culturing on PDA media and incubated at a temperature of 28 °C for 5 days. Then, the fungal discs were added to a potato dextrose broth medium and subjected to static incubation for a period of one week at a temperature of 25 °C. Next, the culture underwent centrifugation at a speed of 10,000 rpm for a duration of 10 min using a Sigma 3–16k centrifuge (manufactured by Sigma Laborzentrifugen GmbH, Osterode am Harz, Germany). This centrifugation process aimed to separate the supernatant from the mycelium. Subsequently, a volume of 1 mL containing a concentration of 3 mM of sodium selenite (Hi-media, Mumbai, India, 99% purity) was introduced into a 10 mL volume of the culture supernatant. The entire reaction mixture was then subjected to incubation at a temperature of 40 °C for a duration of 30 min. Afterward, a detectable reddish coloration appeared, indicating the complete formation of the selenium nanoparticles (SeNPs) [32]. The nanoparticles that were produced were subjected to centrifugation at a speed of 10,000 rpm for 10 min. Following centrifugation, the pellet containing the selenium nanoparticles (SeNPs) was collected. The SeNPs that were produced through myco-fabrication were further purified by being combined with double-distilled water and subjected to three rounds of centrifugation, each lasting 10 min at a speed of 10,000 rpm. The nano-selenium obtained was subjected to a drying process at a temperature of 60 °C for a duration of 48 h, after which it was kept at −20 °C for future utilization.

### 2.3. Characterization of SeNPs

#### 2.3.1. UV-Spectral Analysis

Utilizing a UV-visible spectrophotometer (PerkinElmer Life and Analytical Sciences, CT, OH, USA), the UV-intense absorbances of the nanoparticle solution and the control sample were measured between 400 and 800 nm.

#### 2.3.2. Transmission Electron Microscopy (TEM)

To ascertain the size, form, aggregation, and morphological properties of SeNPs, high-quality pictures were taken using a TEM (HR-TEM; JEOL 2100, Tokyo, Japan).

#### 2.3.3. Dynamic Light Scattering (DLS)

DLS (Zetasizer Nano ZN, Malvern Panalytical Ltd., Worcestershire, UK) was used at an angle equal to 173° and a temperature of 25 °C to determine the particle size and distribution of the SeNPs in terms of the average volume diameters and polydispersity index. DLS was also utilized to determine the zeta potential.

#### 2.3.4. X-ray Diffraction (XRD)

X-ray diffraction (XRD) (Bruker D8 DISCOVER Diffractometer, Billerica, MA, USA) was utilized to determine the crystallinity of the SeNPs. Cu-K radiation with a wavelength of 1.54060 Angstroms was used.

#### 2.3.5. Fourier Transform Infrared Spectroscopy

The possible interactions between the nanoparticles that were synthesized and the metabolites produced by the fungi were determined by analyzing the FTIR absorption spectrum using FTIR spectroscopy (PerkinElmer spectrometer, Akron, OH, USA). In this experimental procedure, a tiny quantity of nanoparticles was subjected to crushing with potassium bromide in order to produce a compact pellet. The examination was conducted within the wavelength range of 400 to 4000 nm.

### 2.4. Biological Applications of SeNPs

#### 2.4.1. Anticancer Effect Evaluation

The MCF-7 breast adenocarcinoma cell line was procured from the Nawah Scientific Laboratory, Cairo, Egypt. In addition to 10% heat-inactivated fetal bovine serum, 100 units/mL of penicillin, and 100 mg/mL of streptomycin, MCF-7 cells were cultured in Dulbecco’s Modified Eagle Medium (DMEM). At 37 °C and 5% carbon dioxide (*v*/*v*), cells were grown in a humidified chamber.

- Sulforhodamine B (SRB) Assay

This assay was utilized to evaluate the cytotoxic effect of SeNPs, as described by [33,34]. Briefly, 100 µL of cell suspension (5 × 10^3^ cells) were spread out among 96-well plates and cultured in the medium for 24 h. The cells were then exposed to a second aliquot of 100 µL of medium containing different concentrations of SeNPs (0.01–100 μg/mL) for 72 h. The medium was changed to contain 150 µL of 10% trichloroacetic acid (TCA), and the cells were incubated at 4 °C for 1 h to fix the cells. After that, the TCA solution was discarded, and the cells were rinsed five times with double-distilled water to ensure complete cleaning. Cells were incubated for 10 min at room temperature in a completely dark environment with 70 µL aliquots of an SRB solution (0.4% *w*/*v* acetic acid) used to wash the plates three times, and then they were left out overnight to dry. Finally, 150 μL of Tris base solution (TRIS) (10 mM) was added, and the absorbance was measured at 540 nm using a BMGLABTEH^®^-FLUOstar Omega microplate reader (Ortenberg, Germany). Cisplatin was utilized as a positive control. The experiment was performed using three replicates.

#### 2.4.2. Antibacterial Activity

The antibacterial potency of SeNPs against pathogenic bacteria was examined using the agar diffusion method. The bacterial strains (*Escherichia coli* ATCC25922, *Pseudomonas aeruginosa* ATCC 7853, *Proteus mirabilis* ATCC29906, and *Klebsiella pneumoniae* ATCC 700721) were cultured in Nutrient Agar (Diffco) medium, which was incubated at 37 °C for 24 h. Following that, a single colony of each strain was inoculated and then cultivated for 24 h at 37 °C in a nutrient broth medium. From each bacterial strain, 100 µL of suspension culture (1 × 10^6^ CFU/mL) was evenly distributed throughout 20 mL of nutritional agar medium. Wells were made in the agar plates using a 6 mm cork borer, and 20 mg/mL of SeNPs were deposited into each well. A negative control was made with a well containing 100 µL of distilled water, while gentamycin (10 μg/disc) was used as the positive control. Inhibition zones formed around the wells were used as a proxy for antibacterial efficacy. Afterward, the size of the inhibitory zones was measured in millimeters. For each bacterial strain, three to five replicates were prepared.

#### 2.4.3. Effect of SeNPs on *Glycine max* Seed Germination and Seedling Growth

*Glycine max* L. seeds were obtained from the Crop Institute at the Agricultural Research Center in Giza, Egypt. Only healthy, uniformly sized seeds were chosen for surface sterilization with an aqueous solution of sodium hypochlorite at a concentration of 1.05% for five minutes. The seeds were then carefully rinsed with distilled water. The seeds were then immersed for 2 h in distilled water (control) or various concentrations of SeNPs solution (0.1, 0.5, 1.0, 2.0, and 3.0 µM). After thoroughly rinsing the seeds with distilled water, they were placed in a 9 cm petri dish that contained Whatman filter paper (No. 2) and was irrigated with 5 mL of distilled water. Seeds were incubated at 25 ± 2 °C with a light period of 16 h. Each treatment comprised four petri dishes containing five seeds each. The experimental design was completely random, and the experiment was repeated three times. After seven days, germinated seeds were cultivated in plant pots with sterilized soil, including sand and peat moss, at a ratio of 1:1. The pots were frequently irrigated with distilled water. The number of germinated seeds was determined after 1, 2, 3, 4, 5, and 6 days following the date of seed planting. The emergence of a radicle manifested the germination of seeds. According to [35,36,37], subsequent germination indices were computed as follows:

Germination percentage (*GP*), which describes the percentage of germinated soybean seeds, was computed according to the following equation:

$$\%GP=\left(Total\;number\;of\;germinated\;seeds\right)/\left(Total\;number\;of\;seeds\right)\;*100$$

Germination energy (*GE*), which demonstrates the rapidity and consistency of seed germination, was calculated using the following equation:

$$\%GE=\left(Number\;of\;germinated\;seeds\;on\;days\;4\right)/\left(Total\;number\;of\;seeds\right)\;*100$$

Germination rate index (*GRI*), which describes the correlation between germination percentage and germination time, was calculated using the following equation:

$$GRI=\sum \left(Gt/Tt\right)$$

In the above equation, *Gt* represents the number of germinated seeds on day *t*, and *Tt* represents the number of days after germination.

Mean germination time (*MGT*), which refers to the average amount of time needed for seeds to germinate at their highest rate, was calculated using the following equation:

$$MGT=\sum \left(Ti*Ni\right)/\sum Ni$$

In the above equation, *Ni* is the total number of newly germinated seeds at time *Ti*.

Root length (RL), lateral root number (RN), shoot length (SL), and seedling fresh weight (FWT) measurements were taken 3 weeks after planting. Then, after drying for 72 h at 50 °C in the oven, the seedlings’ dry weight (DWT) was measured.

#### 2.4.4. Effect of SeNPs on Plant Pathogenic Fungi

The studied fungi, *Fusarium oxysporum*, *Rhizoctonia solani*, and *Fusarium solani* were cultured in potato dextrose agar (PDA) medium for five days at 25 °C. Percentage inhibition of mycelial growth (PIMG) was used to evaluate the antifungal activity against the studied fungi [38]. The following criteria were used to calculate this percentage: As a first step, 20 mg/mL of SeNPs solution was mixed with 20 mL of PDA. Five mm fungal plugs were placed directly into the medium’s center. Sterile PDA media was used as a negative control by inoculating it with agar plugs of the same diameter as those used in the treated plates. A 7-day incubation period at 25 °C was employed for all cultures. To calculate the inhibition percentage of SeNPs (*PIMG*), the radius of mycelium growth on PDA medium containing SeNPs (*R*2) was compared to that of mycelium growth on PDA medium (*R*1) according to the following formula:

$$PIMG=\left\{\left(R1-R2\right)/R1\right\}\times 100.$$

#### 2.4.5. Insecticidal Activity of SeNPs against the Egyptian Cotton Leafworm

The Egyptian cotton leafworm, *S. littoralis*, was reared as egg masses until adulthood. A laboratory-susceptible strain of this pest was obtained from the Plant Protection Research Institute, Agricultural Research Centre (ARC), Egypt. The larvae were fed semi-artificial diet with some modifications [34]. Insects were maintained at optimized laboratory conditions (26 ± 2 °C, 65 ± 10% RH and 8:16 L:D photoperiod) according to [30].

Feeding assay was followed to detect the toxicity of the SeNPs against 4th instar larvae of *S. littoralis*. There were 3 replicates per treatment. Each treatment consisted of 30 4th instar larvae. SeNP concentrations were 5, 10, 20, 30, 40, and 50 mg/mL. A control group was constructed using distilled water only. Larval semi-artificial media (about 4.0 mm) was prepared, 1 mL of either SeNPs or water was added, and the food paste was air-dried. The larvae were starved for at least 3 h, then offered the prepared media for feeding for 24 h. The next day, food was replaced with a non-treated diet. Larval mortality was recorded 48 h post-treatment, and the corrected mortality was calculated [39]. LC_50_ was determined using probit mortality analysis according to [40] using Ldp Line^®^ v. 1 software (http://embakr.tripod.com/ldpline/ldpline.htm (accessed on 1 April 2023)).

### 2.5. Statistical Analysis

Minitab 17 software (accessed in April 2023) was used to run a one-way analysis of variance (ANOVA) on the data that had been collected. When there were significant differences between groups, the Fisher’s Least Significant Difference (LSD) method with a 95% confidence interval was used to compare treatment means.

## 3. Results

### 3.1. SeNPs Characterization

#### 3.1.1. Visual Color

**Figure 1:**
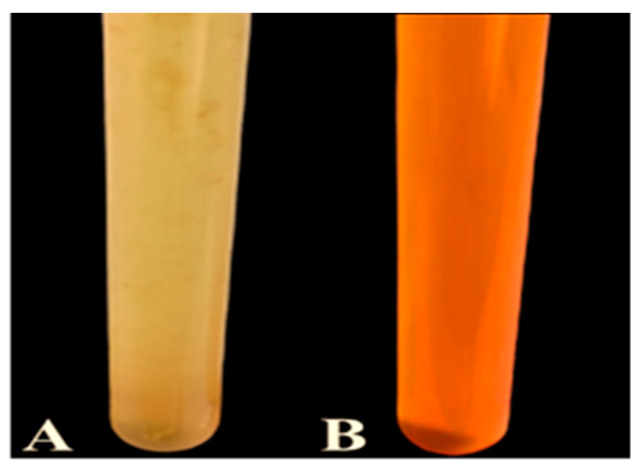
Changing the color of fungal filtrate and sodium selenite from yellow (**A**) to red (**B**) after 30-minute incubation period.

The yellow fungal filtrate changed color to red (Figure 1) after being treated with sodium selenite for 30 min, indicating the creation of nanoparticles.

#### 3.1.2. UV–Visible spectroscopy

**Figure 2:**
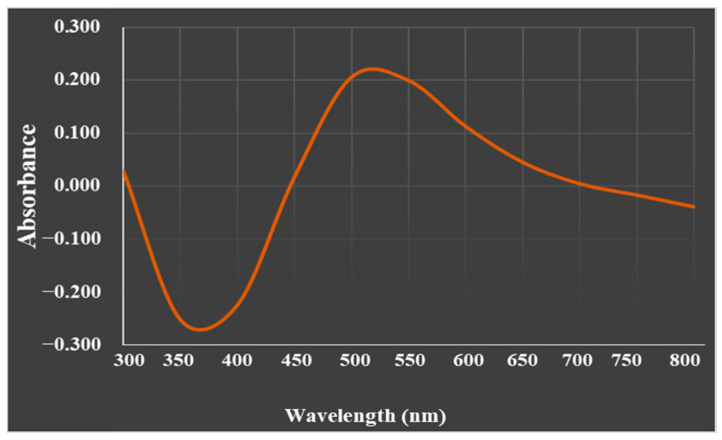
UV–visible spectrum of the biosynthesized SeNPs revealing an SPR peak at 520 nm.

The UV-visible absorption spectrum of the biosynthesized SeNP particles was recorded at 520 nm (Figure 2).

#### 3.1.3. Transmission Electron Microscopy (TEM)

**Figure 3:**
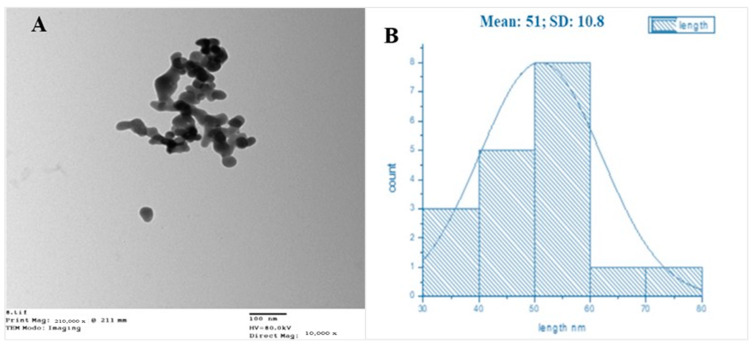
TEM photographs of SeNPs showing the shape (**A**) and size distribution (**B**).

Transmission electron microscopy (TEM) was used to examine the size, shape, morphology, and aggregation of the SeNPs. The results revealed that the particles were evenly dispersed and had a quasi-spherical structure with a diameter ranging from 30 to 80 nm (Figure 3), and a mean average diameter of 51 nm.

#### 3.1.4. Particle Size and Zeta Potential

**Figure 4:**
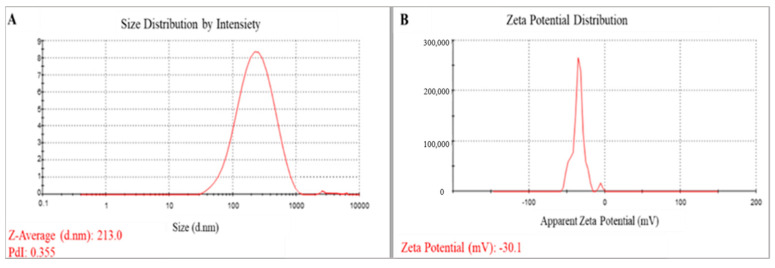
(**A**) Particle size distribution, (**B**) Zeta potential distribution of myco-synthesized SeNPs.

DLS was used to determine the particle size and size distribution as well as the zeta potential of the SeNPs. The average hydrodynamic diameter of the SeNPs was 213 nm (Figure 4A), whereas the average zeta potential was −30.1 mV (Figure 4B). The homogeneity or heterogeneity of the colloidal NPs was assessed using the polydispersity index (PDI) value. In the current investigation, the PDI value was 0.35.

#### 3.1.5. X-ray Diffraction (XRD)

**Figure 5:**
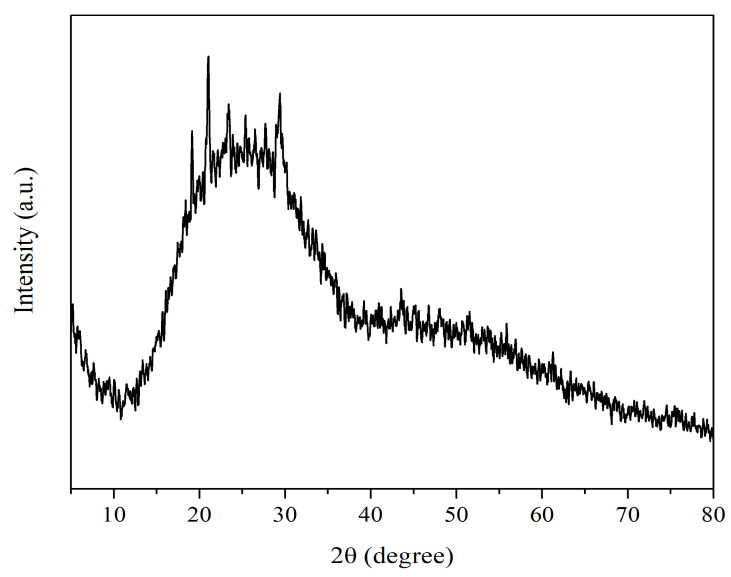
XRD diffraction pattern of myco-synthesized SeNPs. The pattern shows small reflections at 19.14°, 21.01°, 25.35°, and 29.40°, which correspond to elemental selenium (JCPDS 76-1865).

The XRD data showed a broad profile devoid of distinct Bragg peaks. The findings showed that the myco-synthesized SeNPs are less crystalline and largely amorphous (Figure 5). The provided spectrum exhibited minor reflections at angles of 19.14°, 21.01°, 25.35°, and 29.40°, which can be attributed to the presence of elemental selenium (JCPDS 75-1162).

#### 3.1.6. FTIR analysis

**Figure 6:**
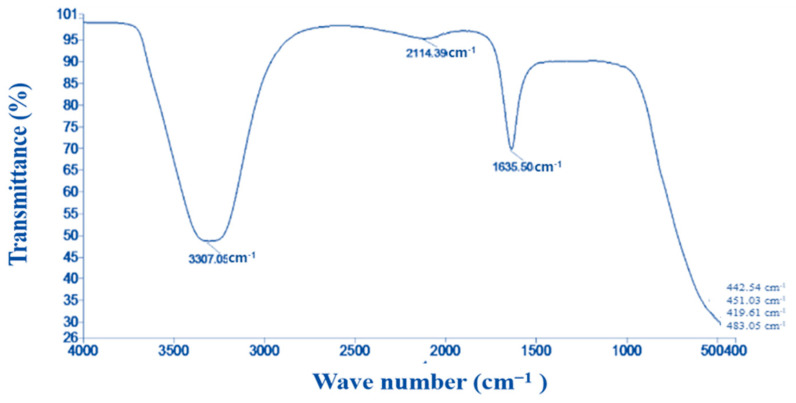
FTIR spectrum of the myco-synthesized SeNPs.

FTIR measurements were used to identify the different functional groups that are present in the metabolites involved in the mycosynthesis, capping, and stabilization of the SeNPs. FTIR analysis of the *P. chrysogenum* culture filtrate revealed five distinct peaks at 3307.57, 2107.89, 1635.22, 431.08, and 407.76 cm^−1^. Eight peaks were added to these peaks in the SeNP spectrum. Wavenumbers at 3307.05, 2114.39, 1635.50, 451.03, 442.54, 429.87, 419.61, and 403.05 cm^−1^ are marked on the spectrum in Figure 6 to demonstrate the interaction of metabolites with SeNPs.

### 3.2. Biological Activities 

#### 3.2.1. Anticancer Activity

**Figure 7:**
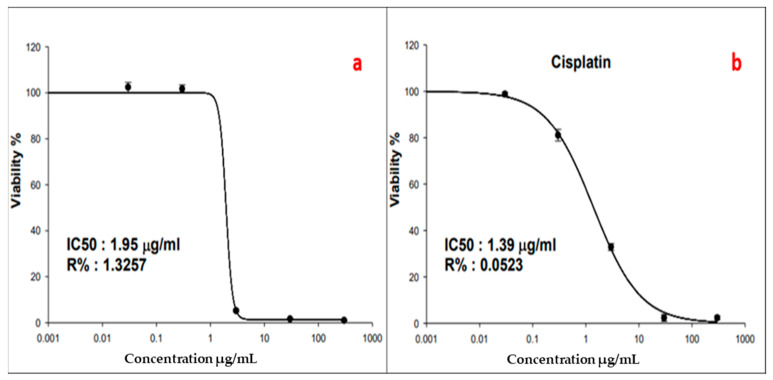
The viability dose-response curve. (**a**) Cultured MCF-7 cells treated with SeNPs, and (**b**) Cultured MCF-7 cells treated with cisplatin, (positive control).

The cytotoxic activity of SeNPs against MCF-7 breast adenocarcinoma cells was determined using the SRB assay. This assay was performed with SeNP concentrations ranging from 0.01 to 100 µg/mL. Cell viability was observed to be dose-dependent (Figure 7). SeNPs revealed cytotoxic activity, and the 50% inhibitory concentration (IC_50_) was determined to be 1.95 µg/mL compared to the IC_50_ (1.39 µg/mL) of the positive control, cisplatin.

**Figure 8:**
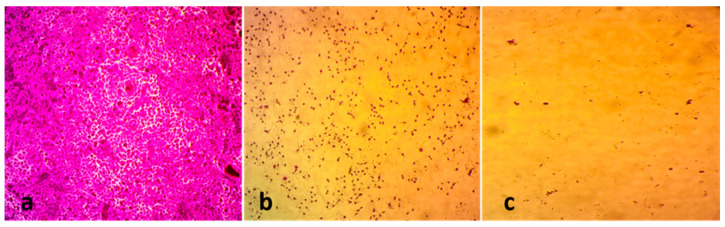
The morphology and viability proliferation and growth of MCF-7 cells. (**a**) MCF-7 cells of negative control, (**b**) MCF-7 cells treated with cisplatin (positive control), and (**c**) MCF-7 cells treated with SeNPs (magnification 100×).

Figure 8 shows the morphological alterations observed in untreated MCF-7 cells, MCF-7 cells treated with cisplatin, and MCF-7 cells subjected to SeNP treatment for a duration of 72 h. After a 72 h treatment period, the examination of MCF-7 cells under a light microscope (Figure 8c) demonstrated that SeNP treatment resulted in a reduction in the number of MCF-7 cells and significant distortion in their morphology, more than the changes observed in cells treated with cisplatin (Figure 8b). The MCF-7 cells that were subjected to treatment exhibited cellular shrinkage and distortion. In contrast, the MCF-7 cells that were not subjected to any treatment had a typical cellular morphology characterized by normal and polygonal cell shapes, as indicated by the presence of a well-defined and discernible cell membrane boundary (Figure 8a).

#### 3.2.2. Antibacterial Activities

**Figure 9:**
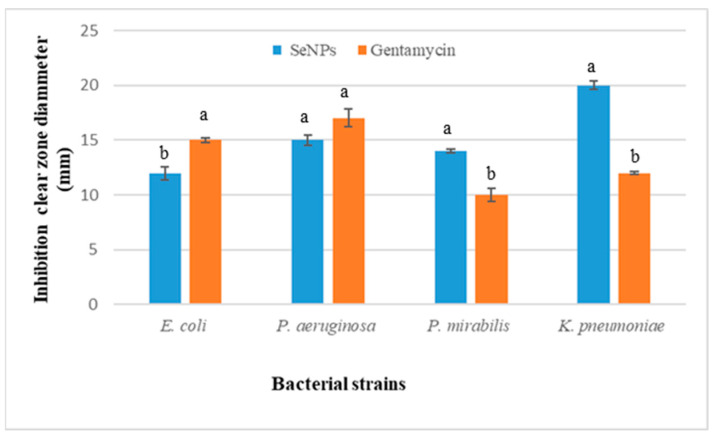
Antibacterial activity of the SeNPs (20 mg/mL) synthesized by *Penicillium chrysogenum* and Gentamycin as a positive control (10 μg/disc). The columns represent the mean values of inhibition zone diameter ± SE. Columns denoted by different alphabetical letters are significantly different at *p* ≤ 0.05.

The antibacterial efficacy of selenium nanoparticles myco-synthesized from *Penicillium chrysogenum* against different pathogenic bacterial strains was investigated. Selenium nanoparticles inhibited the growth of all bacteria tested at 20 mg/mL. SeNPs inhibited *Proteus mirabilis* ATCC29906 and *Klebsiella pneumoniae* ATCC700721 more effectively than the positive control (Figure 9).

#### 3.2.3. Effect of SeNPs on *Glycine max* Seed Germination

**Figure 10:**
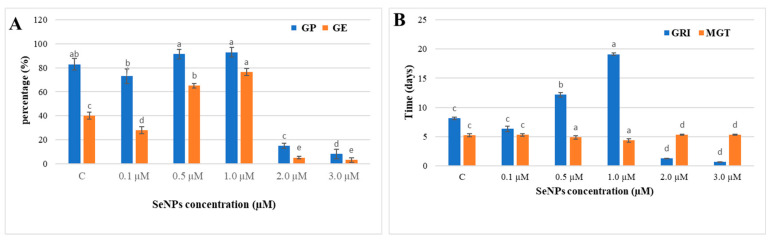
Effect of different concentrations from SeNPs on (**A**) Germination Energy (GE) and Germination Percentage (GP), (**B**) Germination Rate index (GRI), and Mean germination time (MGT) of soybean plant. The columns represent the mean values ± SE. Columns denoted by different alphabetical letters are significantly different at *p* ≤ 0.05.

The effects of different SeNP concentrations (0.0–3.0 µM) on *Glycine max* L. seed germination and seedling growth revealed that, at certain concentrations (i.e., 0.5 and 1.0 µM), SeNPs increased the germination rate, seed germination percentage, and seedling growth rate. The percentage of germinated seeds increased significantly to 93% and 91.5% when treated with 1.0 and 0.5 µM SeNPs, respectively, compared to 83% for the control samples (Figure 10A). With 1.0 µM SeNPs, seed germination was more rapid and uniform, as evaluated by germination energy, germination rate index, and mean germination time values of 76.5%, 19.0, and 4.3 days, respectively. Conversely, the germination was negatively impacted by higher concentrations of SeNPs (2.0 and 3.0 µM), which resulted in low values for GP, GI, and GE (Figure 10A,B).

**Figure 11:**
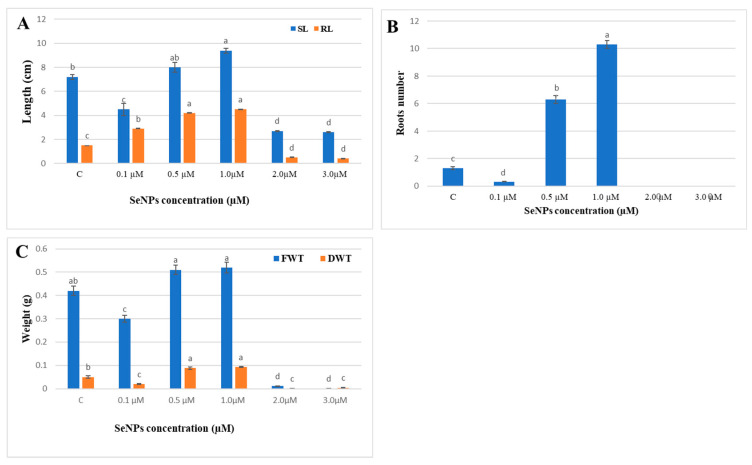
Effect of various SeNP concentrations on (**A**) Root length (RL) and shoot length (SL), (**B**) number of roots (RN), and (**C**) fresh weight (FWT and dry weights (DWT) of soybean seedlings. The columns represent the mean values ± SE. Columns denoted by different alphabetical letters are significantly different at *p* ≤ 0.05.

The effect of SeNP concentrations on seedling growth was comparable to the germination indices of seeds. Using 1.0 µM SeNPs significantly enhanced the shoot length to 9.4 cm and the root length to 4.5 cm, compared to the control values of 7.2 cm and 1.5 cm for shoot and root lengths, respectively. The number of lateral roots increased tenfold when the seeds were soaked in 1.0 M SeNPs in comparison to the control (Figure 11). SeNP concentrations of 0.5 and 1.0 µM resulted in greater fresh weights (0.51, 0.54 g) and dry weights (0.08 and 0.09 g), respectively.

**Figure 12:**
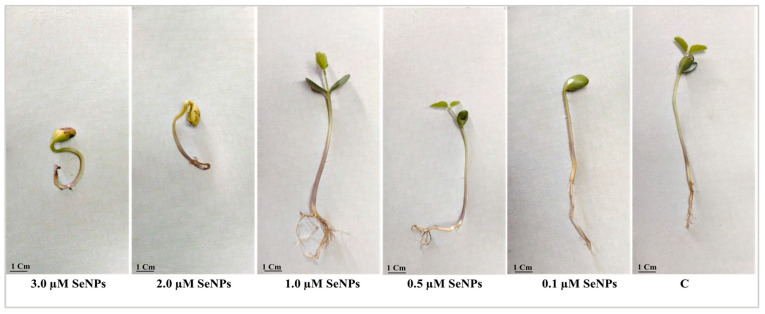
Effect of various SeNP concentrations on the morphological characters of soybean seedlings.

Seeds exposed to 2.0 and 3.0 µM SeNPs failed to germinate or produce leaves or roots, and their cotyledons and radicles appeared yellow to brown (Figure 12).

#### 3.2.4. Antifungal Activities against Plant Pathogens

**Figure 13:**
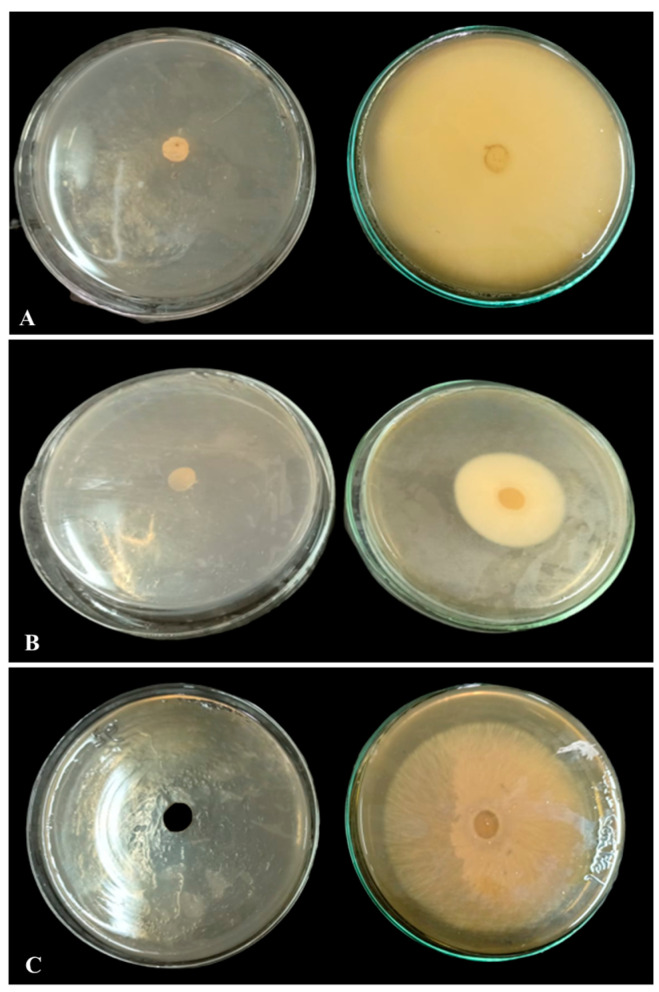
The antifungal activity of SeNPs on the growth rate of (**A**) *Rhizocotonia solani*, (**B**) *Fusarium solani*, and (**C**) *Fusarium oxysporum*.

Three plant pathogens, *Rhizocotonia solani*, *Fusarium oxysporum*, and *Fusarium solani*, were tested for their susceptibility to SeNP antifungal activity, and the results demonstrated a 100% inhibition rate (Figure 13).

#### 3.2.5. Insecticidal Activity

**Figure 14:**
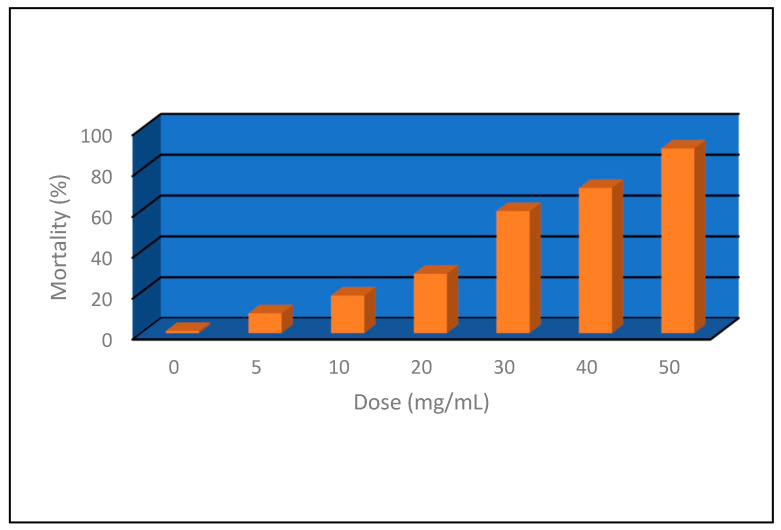
Dose–response assay of the lethal effect of SeNPs on the 4th instar larvae of *S. littoralis*.

The effect of both lethal and sub-lethal doses of SeNPs was investigated in the 4th instar larvae of the Egyptian cotton leafworm, *S. littoralis*, through a dose–response feeding assay. Increasing SeNP concentration administered in a single dose through feeding on semi-artificial media as a larval diet resulted in a significant increase in larval mortality compared to the control group (r = 0.9, *p* ≤ 0.05) (Figure 14). Initially, there was a gradual increase in larval mortality upon treatment with 5.0 up to 20.0 mg/mL SeNPs. Larval mortality was recorded at 9.6 to 29.0%, respectively. A further increase in SeNP dose resulted in a nearly 2.0-fold increase in larval mortality from 29.0 to 60.0%. Probit analysis estimated the median lethal concentration (LC_50_ that killed 50% of the population) as 23.08 mg/mL (Table 1 and Figure 14). Observations on the treated larvae included initial convulsive movements, and cessation of feeding was also observed at higher SeNP concentrations. Larval cadavers showed dryness, and they acquired a black color without any putrefaction odor, assuring the absence of any viral or bacterial contamination as a cause of death or even an incidence of secondary infection, thereby assuring that the main cause of death was the larval food treatment with SeNPs.

## 4. Discussion

Biogenically produced nanomaterials have been used in many fields, including biomedicine, electronics, and agriculture, due to their considerable benefits, including their lack of hazards, low cost, and practicality [41]. In the present investigation, SeNPs were produced using the reducing capability of *P. chrysogenum* culture filtrate metabolites. This study indicated the capability of *P. chrysogenum* metabolites such as proteins, enzymes, and carbohydrate to facilitate the biosynthesis of SeNPs, leading to enhancements in the production process, a reduction in aggregation, and the generation of smaller particle sizes [31]. The alteration in the color of the media filtrate upon its combination with metal precursors served as the initial indication of the biosynthesis of nanoparticles. Different studies used fungal metabolites in SeNP synthesis. Islam et al. [42] effectively conducted the biosynthesis of SeNPs using *Fusarium oxysporum* as the biological agent. SeNPs were synthesized using the process of gamma irradiation in *Monascus purpureus* [43]. A cell-free extract derived from *Aspergillus terreus* was used as a reducing and stabilizing agent during the manufacture of selenium nanoparticles [44]. The colloidal solution containing selenium nanoparticles (SeNPs) was analyzed using UV-visible spectroscopy, revealing the appearance of a red hue at a wavelength of 520 nm. The observed peak may potentially be attributed to the impact of surface plasmon resonance on SeNPs [31,45]. The synthesis of SeNPs utilizing *Acinetobacter* sp. SW30 exhibited two absorption peaks at wavelengths of 300 nm and 500 nm [46]. Furthermore, it was shown that SeNPs formed by gamma-irradiated *Monascus purpureus* exhibited a significant surface plasmon resonance (SPR) peak at a wavelength of 593 nm [43]. The analysis of dynamic light scattering (DLS) is an essential method used to ascertain the dimensions and distribution of nanoparticles (NPs) inside colloidal fluids. The present study utilized DLS analysis to determine the average size of the biosynthesized SeNPs, which was found to be 213 nm. The authors utilized the polydispersity index (PDI) value in order to assess the consistency or heterogeneity of the colloidal nanoparticles [47]. A PDI number below 0.4 indicates a state of high homogeneity, whereas a greater value signifies low homogeneity. Additionally, a PDI value exceeding 1 indicates a heterogeneous solution. In the present investigation, the PDI (Particle Dispersion Index) value was 0.355. The variation in the estimated diameters obtained from dynamic light scattering (DLS) and transmission electron microscopy can be attributed to the different principles underlying these techniques. DLS measures the hydrodynamic size of particles, whereas TEM examines the solid core of particles [48]. The substantial disparity in size seen between DLS and TEM measurements may be due to the aggregation of SeNPs. Potential alterations to the metabolites present on the surface of nanoparticles during the washing and drying procedures may have an impact on the electrosteric barrier. Consequently, this could result in heightened energy levels and reactivity of the nanoparticles, ultimately leading to their aggregation [49].

A zeta potential exceeding +30 mV or falling below +30 mV indicates the electrostatic stability of the colloidal nanoparticle solution [50]. Based on the X-ray diffraction (XRD) results, it was shown that the selenium nanoparticles (SeNPs) synthesized by *P. chrysogenum* had an amorphous structure. The amorphous configuration of selenium nanoparticles seen in this study is consistent with previous research conducted on *Pseudomonas stutzeri* [50,51].

An investigation of the interaction between selenium nanoparticles (SeNPs) and metabolites produced by *Penicillium chrysogenum* has been conducted through the utilization of Fourier Transform Infrared (FTIR) spectroscopy. Based on the N-H, C-H, and O-H stretching vibrations, the signal seen at 3307.05 cm^−1^ indicates the existence of primary amine in the proteins of the fungus [52] and alkyne [53]. This exemplifies the essential role of N-H-containing proteins in the process of synthesizing SeNPs and reducing Se ions. Based on the spectral analysis, it can be inferred that the presence of alkyne is indicated by the band observed at a wavenumber of 2114.39 cm^−1^. Furthermore, the signal observed at a wavenumber of 1635.50 cm^−1^ has been associated with several polysaccharide ring and peptide linkage functional groups, such as N-H, C=N, C=O, and C=C, as reported in [32]. The interaction between selenium nanoparticles (SeNPs) and the metabolites present in the culture filtrate of *P. chrysogenum* was observed through distinct spectral bands at wavenumbers of 451.03, 442.54, 429.87, 419.61, and 403.05 cm^−1^. The results of this study indicated that the capping, stability, and reduction of selenium nanoparticles (SeNPs) are influenced by different functional groups of organic substances present in the culture filtrate of *P. chrysogenum*. These substances include proteins, enzymes, and polysaccharides [31]. 

In the present work, SeNPs were studied for their anticancer potential, specifically against MCF-7 breast adenocarcinoma cells. The presented work demonstrated that SeNPs effectively controlled the growth and proliferation of breast cancer cells. Biogenic SeNPs prepared using *Crataegus monogyna* extracts and *Lactobacillus casei* showed anticancer activity against MCF-7 breast adenocarcinoma cells [53,54]. The anticancer efficacy of SeNPs may be due to their ability to induce apoptosis in cancer cells, which causes cell degeneration and eventual cell death [55,56]. So, the observed morphological alterations in cells treated with SeNPs may potentially be attributed to the induction of apoptosis. Another mechanism by which SeNPs exert their anticancer activity is DNA fragmentation, phosphatidylserine translocation, oxidative stress, and mitochondrial dysfunction [57]. Further studies may be required to determine the exact anticancer mechanism of the myco-synthesized SeNPS.

The results of the present study demonstrated the effectiveness of *Penicillium chrysogenum* myco-synthesized selenium nanoparticles against Gram-negative bacterial strains, demonstrating their potential as an antimicrobial agent. At a concentration of 20 mg/mL, selenium nanoparticles significantly reduced the growth of all tested bacteria. SeNPs were more effective than the positive control at inhibiting the growth of *Proteus mirabilis* and *Klebsiella pneumoniae*. It has been proven that many forms of biogenic selenium produced by fungi have antibacterial capabilities. For instance, the growth of a wide range of bacteria, including *Pseudomonas aeruginosa*, *Escherichia coli*, *Bacillus subtilis*, and *Staphylococcus aureus*, can be suppressed by selenium nanoparticles produced by certain species of *Penicillium*, such as *Penicillium expansum* and *Penicillium crustosum* [13,32]. The permeability of bacterial cell membranes is altered and damaged by biogenic SeNPs, which cause polysaccharides and proteins to leak out of the cells and ultimately result in cell death [58]. Additionally, SeNPs cause oxidative damage to bacterial cells [58]. SeNPs have been proven to impair cellular respiration and decrease transmembrane potential by affecting the integrity of the cell membrane and blocking the function of enzymes crucial to bacterial growth and metabolism, such as dehydrogenases [59].

In the present work, a study of the effects of SeNPs at varying concentrations (0.0–3.0 µM) on soybean seed germination revealed that concentrations of 1.0 and 0.5 µM significantly enhanced all measured germination assessments, including germination energy, germination percentage, germination index, and reduced mean time of germination. Also, 1.0 µM SeNPs enhanced shoot and root length and weight and dramatically increased the number of lateral roots compared to control samples. At the same time, our results showed that higher concentrations of SeNPs (2.0 and 3.0 µM) showed a negative impact on all germination assessments and seedling growth. Seed germination is a long and intricate biological process that begins with the dry seed absorbing water and ends when the radicle pushes through the seed coat [60]. Successful germination of soybean seed is the first step in increasing the plant’s yield and improving its quality. Results from our study agree with recent studies on other plant species showing that SeNPs, at low concentrations, improve seed germination and boost plant growth. For example, wheat plants treated with selenium nanoparticles had significantly longer shoots and roots, higher fresh and dry weights, more leaves, and higher leaf lengths; however, larger concentrations decreased plant development [61]. Also, Garza-Garca et al. [62] reported that the effect of selenium nanoparticles on plant growth depends on several factors, such as the concentration of the selenium nanoparticles and the method used to synthesize them. Selenium nanoparticles have been shown to improve seed germination, shoot length, and root length in Hordeum vulgare [63], enhance root growth in lettuce plants [64], improve groundnut plant shoot and root length and weight [65], and enhance growth in *Helianthus annuus* [52]. Selenium nanoparticles promote seed germination by increasing the amount of soluble amino acids and soluble sugars and boosting the activity of seed-germination enzymes like α-amylase, β-amylase, and proteases [66].

In the present work, utilization of SeNPs produced from *Penicillium chrysogenum* has shown zero growth in the three studied fungi: *Rhizocotonia solani*, *Fusarium oxysporum*, and *Fusarium solani*. These are plant pathogenic fungi that cause damage and yield loss in a variety of plant species. *Rhizocotonia solani*, for example, causes hypocotyl and root rot as well as leaf blight in soybean plants [67]. *Fusarium* species are well known as the major causative agent of root rot disease, which infects soybean plants in many places across the world [68]. Different studies have reported that selenium nanoparticles produced by different strains of fungi like *Aspergillus flavus* and *Candida albicans* are effective at inhibiting the growth and proliferation of different *Candida* species [69,70]. Also, selenium nanoparticles produced by *Trichoderma atroviride* showed antifungal activity against *Pyricularia grisea*, *Colletotrichum capsici*, and *Alternaria solani* [69,71]. Nanoparticles’ antifungal activity is caused by their ability to disrupt cell membranes and cell walls, which ultimately results in fungal cell death [11,72].

In terms of their insecticidal effect, the toxic effect of green-synthesized SeNPs on aquatic insects, such as the Egyptian yellow fever mosquito, *Aedes egypti*, showed a high susceptibility to low SeNP concentrations of 10 µg/mL, recording 99% mortality [72]. Moreover, *Aedes albobictus* mosquitoes showed dose-dependent mortality, supporting our findings about its possible application against insects. A concentration range of 10 to 50 mg/mL was also effective against mosquito larvae, attaining mortality levels ranging from about 28 to 82% [73]. Considering median lethal concentrations (LC_50_), *Culex pipiens larval* treatment with plant-based selenium nanoparticles resulted in 50% population mortality at a hypothetical concentration of 28.8 mg/mL of SeNPs for the 4th instar larvae, which is considered the least susceptible instar during the larval stage [74]. Variations in the insecticidal properties of plant-derived selenium nanoparticles may exist due to variations in plant secondary metabolites that act as stabilizing and capping agents, as well as other phytochemicals that may have insecticidal effects. Those variations could be a reason for differences in insect responses to numerous, green-synthesized selenium nanoparticles [75]. The inevitable application of nanoparticles during agricultural practices such as fertilization, fungicide, and pesticide application, etc. may lead to tremendous effects on humans, plants, and animals, including many insect species. Field application of green synthesized SeNPs significantly reduced the cutworm (*Agrotis ipsilon*) larval population at a rate of 25 ppm; the same concentration had a growth-promoting effect on the host plant [52]. Having a plant-growth-promoting effect as well as fighting major insect pests is advantageous in addressing the challenge of reducing chemical insecticide application while also increasing plant yield. The toxicity of nanoparticles may rely on impairing normal cellular activities as well as disrupting the structural integrity of the genetic material, enzymes, and oxidative stress, leading to cell death [52].

## 5. Conclusions

Biogenic SeNPs synthesized by *P. chrysogenum* exhibit multiple biological activities. They exhibited anticancer activity against MCF-7 breast adenocarcinoma cells as well as antibacterial activity against four human pathogens. The addition of SeNPs at low concentrations was observed to stimulate the growth of soybean plants by enhancing seed germination and supporting the development of shoots and roots. In addition, SeNPs increased the mortality rate of *S. littoralis*, the Egyptian cotton leafworm larvae, and inhibited the growth of the plant pathogenic fungi *Rhizocotonia solani*, *Fusarium oxysporum*, and *Fusarium solani*. The activities of SeNPs reported in this study are significant in medicinal and agricultural applications for boosting crop yield while lowering the negative environmental impact of chemical anticancer, antibacterial, insecticide, and fertilizer agents.

## Figures and Tables

**Table 1:** Probit analysis of the lethal effect of SeNPs on 4th instar larvae of *S. littoralis*.

Lethal Conc. (LC)	Conc. (mg/mL)	95% Fiducial Limits (mg/mL)		Slope	Probability (P)	Regression Analysis (R^2^)
		Lower	Upper			
25 50 90	12.36 23.08 75.66	6.49 15.57 66.56	15.41 34.02 188.95	2.49 +/− 0.12	0.0004	0.95

## Data Availability

All data generated or analyzed during this study are included in this article.
